# Evaluating Maternal and Fetal Risk in Preeclampsia Using Thyroid and Inflammatory Markers: A Naive Bayes and Network Analysis Approach

**DOI:** 10.7759/cureus.115127

**Published:** 2026-08-24

**Authors:** Prakruti Dash, Saurav Nayak, Tanushree Roy, Bharath Kumar Koppisetty, Kasala Farzia, Vinodini Kadir

**Affiliations:** 1 Biochemistry, All India Institute of Medical Sciences, Bhubaneswar, Bhubaneswar, IND; 2 Biochemistry, Indian Council of Medical Research (ICMR) National Institute of Child Health Research, New Delhi, IND; 3 Biochemistry, Apollo Institute of Medical Sciences and Research, Chittoor, IND; 4 Obstetrics and Gynecology, All India Institute of Medical Sciences, Bhubaneswar, Bhubaneswar, IND

**Keywords:** anti-tpo antibodies, machine learning in medical diagnosis, naive bayes model, predictive biomarkers, preeclampsia integrated estimate of risk, thyroid-stimulating hormone

## Abstract

Background: Preeclampsia is a significant factor in maternal and fetal morbidity globally, highlighting the necessity for early risk assessment and intervention. This study introduces a machine learning (ML) approach that integrates maternal thyroid profiles and C-reactive protein (CRP) levels to predict preeclampsia and potential fetal thyroid abnormalities.

Materials and methods: A cross-sectional dataset of 174 pregnancies (91 normal, 83 preeclampsia) was analyzed. EN regularization identified maternal CRP, thyroid-stimulating hormone (TSH), and anti-thyroperoxidase antibody as the primary predictors of preeclampsia, with CRP being the most significant contributor.

Results: A Naive Bayes model trained on these markers achieved remarkable predictive performance, with an accuracy of 92.5% ± 7.7%, a sensitivity of 99.9%, and a specificity of 86.1% ± 1.4%. Network analysis identified a substantial maternal-fetal connection via FT3 and cord TSH, establishing the foundation for a secondary NB model predicting fetal hypothyroidism. The model exhibited moderate overall accuracy (63.7% ± 5.3%) but demonstrated significant improvement in the preeclampsia subgroup (73.3% ± 1.7%) while maintaining 100% sensitivity. CRP levels differed significantly between groups, underscoring its role as a systemic inflammatory marker in the pathogenesis of preeclampsia.

Conclusions: This comprehensive ML architecture links maternal indicators to fetal outcomes, demonstrating the practicality of using interpretable, probabilistic models in prenatal care. The results support early risk classification and targeted monitoring for high-risk pregnancies, emphasizing the clinical value of integrating thyroid and inflammatory markers into maternal screening. Future validation across varied cohorts may facilitate the clinical implementation of these models to enhance maternal-fetal health outcomes.

## Introduction

The National High Blood Pressure Education Program of the National Heart, Lung, and Blood Institute (NHLBI) categorizes hypertensive disorders of pregnancy as follows: chronic hypertension, gestational hypertension, preeclampsia, and preeclampsia superimposed on preexisting hypertension [1]. Preeclampsia affects multiple maternal systems, with a global incidence of approximately 2-4% and a high rate of severe complications ranging from 5-20% [2]. It is characterized as a cumulative syndrome marked by the new onset of hypertension (systolic blood pressure >140 mm Hg and diastolic >90 mm Hg on two separate occasions at least four hours apart, or in severe instances, systolic blood pressure >160 mm Hg and diastolic blood pressure >110 mm Hg) and proteinuria (protein (mg)/creatinine (mg) ratio >0.3 or protein >5 g in a 24-hour urine sample, or >3 g in two samples collected six hours apart from a patient at rest) occurring after 20 weeks of gestational age in pregnant women, which resolves by six weeks postpartum [3]. In India, the prevalence ranges from about 6.2% to 18.6%, and 7-8% of maternal deaths are directly associated with hypertensive disorders of pregnancy [4-6].

The demonstrated influence of thyroid function on maternal and fetal outcomes highlights the need for a thorough understanding of its dynamics. Changes in thyroid physiology commence with the onset of pregnancy, continue during gestation, and are reversible after childbirth. The significant fluctuations in thyroid hormone levels during pregnancy, as evidenced by reversible alterations in the thyroid gland, are particularly notable [7]. Women with both overt and subclinical thyroid disorders face an elevated risk of pregnancy-related problems. Fetal complications encompass low birth weight, first trimester spontaneous abortions, preterm delivery, fetal or neonatal hyperthyroidism, intrauterine growth restriction, elevated rates of stillbirth and neonatal mortality, neonatal hyperbilirubinemia, a higher incidence of neonatal hypothyroidism, and increased perinatal mortality [8]. Elevated thyroid-stimulating hormone (TSH) levels have been linked to heightened systolic blood pressure and diastolic blood pressure in non-pregnant individuals. Pregnancy-specific physiology results in variations in TSH, free tetraiodothyronine (FT4), and anti-thyroperoxidase antibody (ATPO) concentrations compared to the non-pregnant condition. ATPO positivity may diminish the thyroid's sensitivity to HCG during pregnancy and affect the TSH cutoff value. Disregarding ATPO during investigation may lead to an underestimation of the actual impact of TSH or FT4 on HDP during pregnancy. Moreover, thyroid function changes during pregnancy, and the timing of thyroid assessments in late gestation may contribute to the variability observed in prior research findings [9].

Another determinant of preeclampsia is profound endothelial disruption and heightened systemic inflammatory activation. This disruption is evident in the atypical expression of inflammatory markers, such as C-reactive protein (CRP), an acute-phase reactant that is significantly elevated during viral illnesses and inflammation [10]. Considering the varied multifactorial conditions associated with preeclampsia and fetal outcomes, robust machine learning (ML) algorithms may therefore be useful for predicting as early as possible the probability of pregnant women developing complications. The relationship between maternal parameters and fetal outcomes is also important because it may influence neonatal health. Given the limited research in this area and the resulting lack of clinical application, we have developed an accurate predictive model for maternal preeclampsia and fetal outcomes using ML models alongside maternal blood thyroid profiles and CRP.

## Materials and methods

Study design

The study was designed as a hospital-based, cross-sectional study conducted in the Department of Biochemistry, in collaboration with the Department of Obstetrics and Gynecology, over a period of one year, from April 2022 to March 2024, after obtaining clearance from the Institutional Ethics Committee of All India Institute of Medical Sciences, Bhubaneswar (approval number: T/IM-NF/Biochem/21/86, approval date: November 8, 2021). Recruitment, sample collection, and laboratory analysis were all completed within this study period.

Sample size

The total sample size was estimated based on the severe complication of maternal mortality due to preeclampsia, which was 8%. With a 95% alpha level and 80% power, the total sample size was estimated at 151. With a 15% attrition rate, the final sample size was 176.

Study participants

Pregnant females who volunteered to participate were included in the study. The final sample size analyzed was 174, following the post-enrollment withdrawal of two participants. Consequently, 91 females with normal pregnancies and 83 with preeclampsia were included in the final analysis. Any patient already diagnosed with and being treated for a thyroid disorder or any other chronic disease condition was duly excluded from the study.

Gestational alignment

Pregnant females evaluated at >29 weeks of gestation were invited to participate. Preeclampsia was diagnosed according to established NHLBI/ACOG clinical criteria, and all cases comprised early-onset presentations (<34 weeks of gestation). To minimize gestational confounding, normotensive control participants were gestational-age-matched to the preeclampsia cohort at recruitment.

Maternal blood sampling

For the preeclampsia group, 5 mL of maternal blood was drawn at the time of clinical diagnosis, prior to the administration of medical therapy. Corresponding maternal samples for the normotensive control group were collected during routine third-trimester antenatal visits at a comparable gestational age (>29 weeks). Cord blood samples were obtained immediately post-delivery for both groups as per protocol.

Estimation of thyroid profile and CRP

TSH, FT4, and free triiodothyronine (FT3) were measured using the Roche Cobas e801 immunoassay by the eCLIA method. ATPO was also measured similarly. CRP was measured on a Beckman Coulter AU5800 system (Beckman Coulter, Inc., CA, USA) by the latex agglutination turbidimetry method.

ML Naive Bayes model

Elastic net (EN) regularization was applied to all parameters to identify which parameters were important for predicting preeclampsia from the maternal profile. Following this, a Naive Bayes model was used to perform classification.

ML network analysis

A symbolic rule engine was implemented to extract interpretable relationships among discretized maternal and fetal biomarkers in the study of preeclampsia. Data preprocessing included binning continuous variables (maternal and cord blood levels of CRP, TSH, FT4, FT3, and ATPO) into categorical labels (e.g., low, normal, high), followed by label encoding for model compatibility. A directed network was constructed based on hypothesized or biologically plausible edges connecting maternal and fetal variables, reflecting thyroid-immune interactions.

Prediction of fetal hypothyroidism

Based on the network analysis results, a probabilistic model was developed to estimate the risk of fetal hypothyroidism from maternal parameters.

Evaluation metrics

All evaluations were performed by 10-fold bootstrapped cross-validation. Accuracy, sensitivity, specificity, positive predictive value, and negative predictive value were measured and reported as mean ± standard deviation (SD).

Statistical analysis

All statistical analyses were carried out in SPSS Statistics version 26.0 (IBM Corp., Released 2019. IBM SPSS Statistics for Windows, Version 26.0. Armonk, NY) and in Python, with the random seed fixed at 1729 for all resampling and cross-validation procedures to ensure reproducibility. Continuous variables were expressed as mean ± SD and categorical variables were expressed as frequencies and percentages. Continuous variables were compared between the preeclampsia and normal pregnancy groups using the two-tailed independent-samples t-test, with degrees of freedom calculated as n₁ + n₂ − 2 = 172; the corresponding effect size was quantified as Cohen’s d computed on the pooled SD and interpreted as small (0.2), medium (0.5), or large (0.8). Categorical variables, namely the proportion of neonates with elevated cord blood TSH, were compared using the Pearson chi-square test with Yates’ continuity correction, and the phi coefficient (φ) was reported as the effect size for the 2 × 2 comparison. For each test, the test statistic, degrees of freedom, p-value, and effect size are reported together in the relevant table. EN regularization was used for feature selection and the Naive Bayes classifier for prediction, with discrimination metrics (accuracy, sensitivity, specificity, PPV, and NPV) obtained from 10-fold bootstrapped cross-validation and expressed as mean ± SD. A two-tailed p < 0.05 was pre-specified as the level of statistical significance for all comparisons.

## Results

The study comprised 174 pregnancies, 91 normal and 83 diagnosed with preeclampsia, in whom TSH, FT3, FT4, ATPO, and CRP were measured in both maternal serum and fetal cord blood. The reference intervals for these analytes in maternal serum and cord blood, along with their units, are given in Table 1. The cord blood intervals are consistently wider and shifted upward relative to the maternal intervals, most markedly for TSH (0.7-13.2 versus 0.4-8.9 mIU/L) and CRP (3-61 versus <10 mg/L), and these compartment-specific limits were used throughout for classifying each measured value as low, normal, or high.

**Table 1:** Reference ranges for analytes measured in maternal serum and cord blood CRP: C-reactive protein, TSH: thyroid-stimulating hormone, FT4: free tetraiodothyronine, FT3: free triiodothyronine, ATPO: anti-thyroperoxidase antibody

Parameter	Maternal range	Cord blood range	Units
CRP	<10	3-61	mg/L
TSH	0.4-8.9	0.7-13.2	mIU/L
FT4	0.8-2.7	2.2-5.3	ng/dL
FT3	2.1-4.4	0.15-3.91	pg/mL
ATPO	<30	<35	IU/mL

The two groups were comparable in maternal age (26.11 ± 5.10 versus 27.10 ± 5.15 years; t(172) = −1.271, p = 0.204, Cohen’s d = −0.193), so the comparisons that follow are unlikely to be confounded by age. Among the maternal analytes, only CRP differed significantly between the groups, and the magnitude of the difference was large: preeclamptic women had approximately sixfold higher concentrations than normotensive women (35.31 ± 21.54 versus 5.91 ± 4.08 mg/L; t(172) = 12.775, p < 0.001, d = 1.939). Maternal ATPO, FT3, FT4, and TSH levels did not differ significantly between groups, with negligible to small effect sizes (|d| ≤ 0.31). However, both maternal FT4 and TSH were slightly lower in preeclampsia and approached the significance threshold (p = 0.055 and p = 0.051, respectively).

In cord blood, CRP was again markedly higher in the preeclampsia group (24.59 ± 14.92 versus 6.58 ± 4.06 mg/L; t(172) = 11.075, p < 0.001, d = 1.681), and cord FT4 was significantly lower (1.17 ± 0.23 versus 1.38 ± 0.22 ng/dL; t(172) = −6.213, p < 0.001, d = −0.943), whereas cord ATPO, FT3, and TSH showed no significant difference. Taken together, this pattern indicates that the systemic inflammatory signal of preeclampsia is mirrored in the fetal compartment and is accompanied by a reduction in fetal free T4, while mean fetal TSH remains unchanged. The full set of comparisons, with the test statistic, degrees of freedom, p-value, and effect size for each analyte, is presented in Table 2.

**Table 2:** Comparison of analytes measured in maternal and cord blood # Groups were compared using the two-tailed independent samples t-test (preeclampsia n = 83, normal pregnancy n = 91; degrees of freedom = 172). Values are mean ± SD. t is the test statistic, and Cohen’s d is the effect size, interpreted as small (0.2), medium (0.5), and large (0.8). A p-value < 0.05 was pre-defined as the level of statistical significance. CRP: C-reactive protein, TSH: thyroid-stimulating hormone, FT4: free tetraiodothyronine, FT3: free triiodothyronine, ATPO: anti-thyroperoxidase antibody, SD: standard deviation

Parameter	Overall (mean ± SD)	Preeclampsia	Normal pregnancy	t (df = 172)	p-value^#^	Cohen's d
Age	26.63 ± 5.13	26.11 ± 5.10	27.10 ± 5.15	-1.271	0.204	-0.193
Maternal CRP	19.93 ± 21.10	35.31 ± 21.54	5.91 ± 4.08	12.775	<0.001	1.939
Maternal ATPO	47.76 ± 60.23	47.56 ± 87.41	47.94 ± 3.56	-0.040	0.968	-0.006
Maternal FT3	3.28 ± 0.62	3.25 ± 0.88	3.30 ± 0.19	-0.474	0.648	-0.072
Maternal FT4	1.30 ± 0.29	1.26 ± 0.38	1.35 ± 0.14	-2.010	0.055	-0.305
Maternal TSH	2.94 ± 1.28	2.74 ± 1.54	3.13 ± 0.96	-2.016	0.051	-0.306
Cord blood CRP	15.17 ± 13.98	24.59 ± 14.92	6.58 ± 4.06	11.075	<0.001	1.681
Cord blood ATPO	21.55 ± 32.37	22.67 ± 40.39	20.52 ± 22.91	0.435	0.670	0.066
Cord blood FT3	2.46 ± 0.74	2.51 ± 0.70	2.41 ± 0.78	0.870	0.384	0.132
Cord blood FT4	1.28 ± 0.25	1.17 ± 0.23	1.38 ± 0.22	-6.213	<0.001	-0.943
Cord blood TSH	7.36 ± 5.62	7.18 ± 5.60	7.52 ± 5.66	-0.389	0.695	-0.059

**Figure 1:**
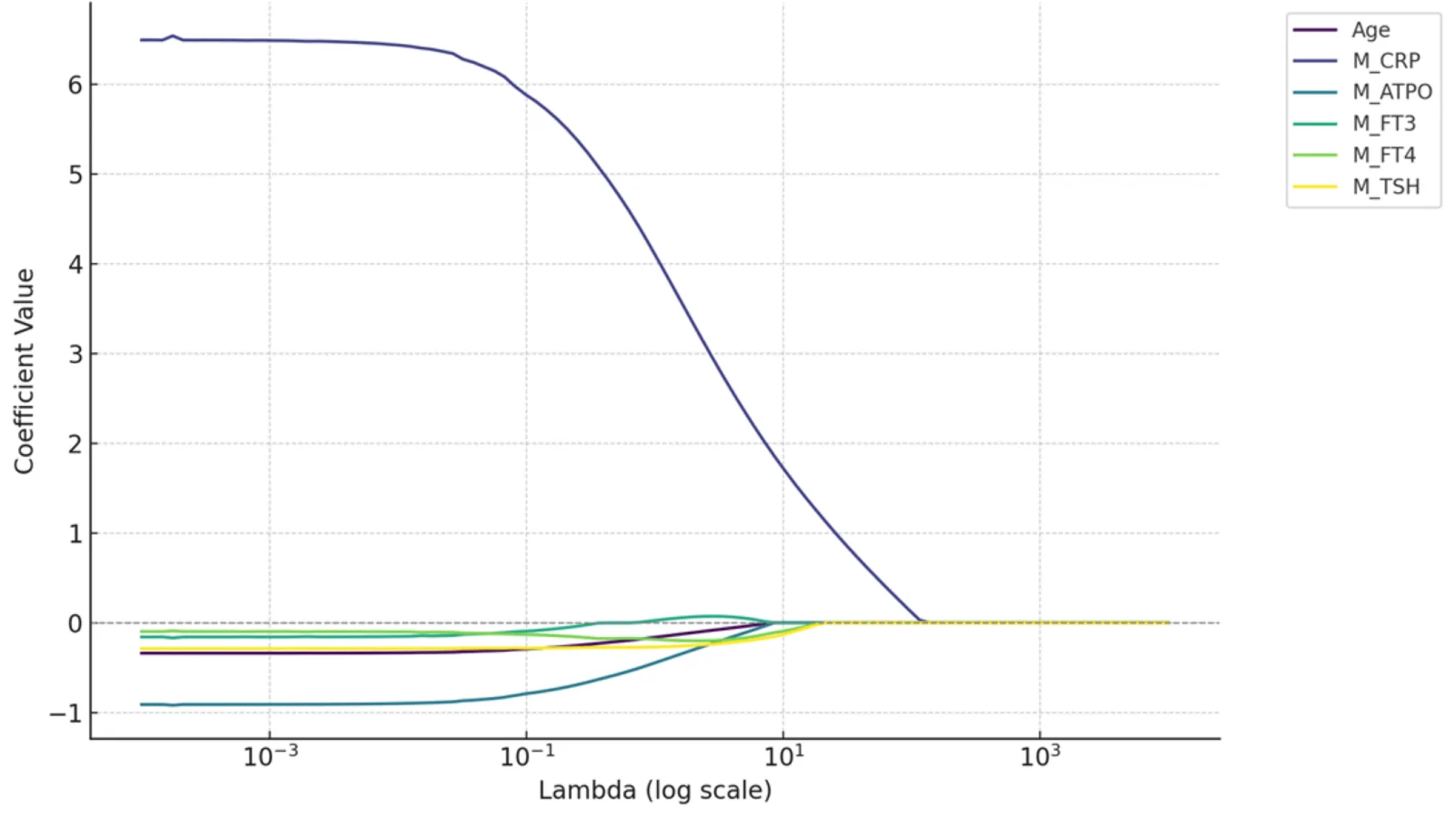
EN coefficient path for maternal parameters for predicting preeclampsia M: maternal, CRP: C-reactive protein, TSH: thyroid-stimulating hormone, FT4: free tetraiodothyronine, FT3: free triiodothyronine, ATPO: anti-thyroperoxidase antibody, EN: elastic net

EN regularization ranked maternal CRP, ATPO, and TSH as the three strongest predictors of preeclampsia. As detailed in Table 3, CRP carried a large positive coefficient (4.732) and alone accounted for 78.65% of the relative importance of the model, whereas ATPO (−0.573; 9.52%), TSH (−0.287; 4.76%), age (−0.215; 3.57%), and FT4 (−0.196; 3.25%) contributed negatively and only modestly, and FT3 was effectively uninformative (0.014; 0.24%). The coefficient path in Figure 1 illustrates the same relationship across the regularization spectrum: the CRP coefficient enters the model first. It remains substantially nonzero throughout the path, whereas the coefficients of the thyroid parameters and age remain close to zero and shrink to zero at moderate penalty strengths. CRP is therefore both the dominant and the most stable predictor of preeclampsia in this cohort, with the maternal thyroid markers adding only marginal, negatively directed information.

**Table 3:** Coefficients and relative importance of parameters for predicting preeclampsia using EN CRP: C-reactive protein, TSH: thyroid-stimulating hormone, FT4: free tetraiodothyronine, FT3: free triiodothyronine, ATPO: anti-thyroperoxidase antibody, EN: elastic net

Rank	Feature	Coefficient	Direction	Relative importance (%)
1	CRP	4.732	Positive	78.65
2	ATPO	-0.573	Negative	9.52
3	TSH	-0.287	Negative	4.76
4	Age	-0.215	Negative	3.57
5	FT4	-0.196	Negative	3.25
6	FT3	0.014	Positive	0.24

The NB model utilizing CRP, TSH, and ATPO, trained and tested on the dataset, showed very high predictive power. The model distinguished preeclamptic from normal pregnancies with an accuracy of 92.5% ± 7.7%. With a sensitivity of 99.9% and a specificity of 86.1% ± 1.4%, the model proved highly effective in predicting preeclampsia.

**Figure 2:**
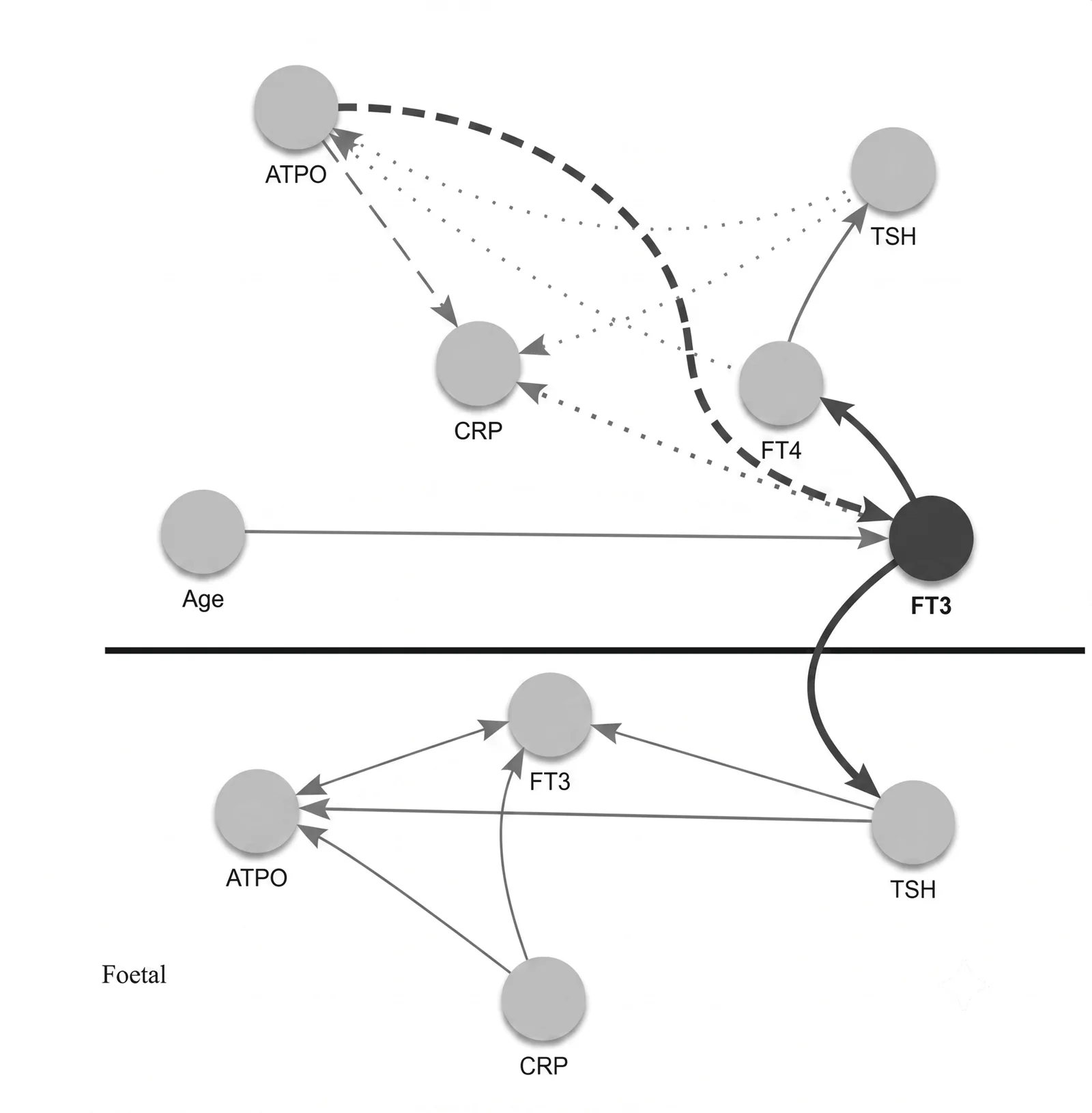
ML network analysis showing relationships among parameters and the maternal–fetal bridging connection CRP: C-reactive protein, TSH: thyroid-stimulating hormone, FT4: free tetraiodothyronine, FT3: free triiodothyronine, ATPO: anti-thyroperoxidase antibody Image source: network analysis figure generated from the study data

The directed network linking maternal and cord blood biomarkers is shown in Figure 2, in which solid edges denote positive relationships and dotted edges denote negative ones. The maternal biomarkers formed one cluster, and the cord blood biomarkers another, and the two compartments were connected by a single bridging edge running from maternal FT3 to cord blood TSH. Because this was the only edge crossing the maternal-fetal boundary, maternal FT3 was identified as the sole candidate maternal predictor of fetal thyrotropin status, and this relationship formed the basis of the probabilistic model of apparent fetal hypothyroidism described below.

Elevated cord blood TSH, defined as a concentration above the upper cord blood reference limit of 13.2 mIU/L, was present in 9.25% of the cohort overall. The proportion was almost identical in normal pregnancies (8.79%) and in preeclampsia (9.63%), and the difference was neither statistically significant nor of any practical magnitude (χ²(1) = 0.000, p = 1.000, φ = 0.015); the comparison is summarized in Table 4. Preeclampsia therefore did not, by itself, alter the frequency of biochemical fetal hypothyroidism at the categorical level, which is why prediction was instead conditioned on maternal FT3 via the bridging relationship identified in the network analysis.

**Table 4:** Comparison of the proportion of neonates with elevated cord blood TSH between preeclampsia and normal pregnancies Values are n (%). Groups were compared using the Pearson chi-square test with Yates’ continuity correction; df = degrees of freedom, φ = phi coefficient (effect size for a 2 × 2 table, where 0.1 is small, 0.3 medium, and 0.5 large). A p-value < 0.05 was predefined as the level of statistical significance. TSH: thyroid-stimulating hormone

Cord blood TSH category	Preeclampsia (n = 83)	Normal pregnancy (n = 91)	χ² (df)	p-value	Effect size (φ)
Elevated (>13.2 mIU/L)	8 (9.63%)	8 (8.79%)	0.000 (1)	1.000	0.015
Within reference (≤13.2 mIU/L)	75 (90.37%)	83 (91.21%)	-	-	-

The Naive Bayes model predicting elevated cord blood TSH from maternal FT3 achieved an accuracy of 63.7% ± 5.3% in the full cohort, with 100% sensitivity and 27.4% specificity. Within the preeclampsia subgroup, accuracy rose to 73.3% ± 1.7%, with sensitivity maintained at 100% and specificity improving to 46.7%. The maternal FT3-cord TSH relationship is thus appreciably stronger in preeclamptic pregnancies, and the model detects every affected neonate at the cost of a considerable false-positive rate, a trade-off appropriate to a screening rather than a diagnostic application.

## Discussion

This study developed a predictive model for preeclampsia and fetal outcomes using ML algorithms that incorporate maternal blood thyroid profiles and CRP levels. Our findings underscore the effectiveness of a Naive Bayes model incorporating maternal CRP, TSH, and ATPO levels, which exhibits substantial predictive capability for preeclampsia. The classification of a pregnancy as preeclamptic versus normal pregnancies showed an accuracy of 92.5% ± 7.7%. The model had a sensitivity of 99.9% and a specificity of 86.1% ± 1.4%, indicating its high predictive performance. Numerous studies have investigated ML models for predicting preeclampsia, typically to enhance early diagnosis and risk stratification [11,12]. The high sensitivity of our model is particularly advantageous, since it reduces the likelihood of false negatives, ensuring that a significant percentage of preeclampsia cases are detected.

In evaluating the physiological underpinnings of our predictive model, the retention of maternal TSH and ATPO, alongside CRP, by EN regularization aligns with emerging evidence of gestational immune-endocrine dysregulation. During normal gestation, placental hCG weakly stimulates TSH receptors to increase thyroid hormone synthesis. However, in autoimmune states characterized by ATPO positivity, autoantibodies can blunt the hCG-mediated response, thereby altering normal gestational TSH thresholds [7]. While univariate analysis in our cohort showed non-significant baseline differences in maternal TSH (p = 0.051) and ATPO (p = 0.968), their multivariable contributions in the Naive Bayes model indicate that thyroid autoantibodies act as subtle risk-modifying covariates rather than as standalone primary drivers. Unlike studies in non-pregnant populations or unselected cohorts, where elevated TSH correlates directly with systemic vascular resistance [9], or elevated ATPO levels predominate independently [13], our ML framework demonstrates that ATPO and TSH provide critical synergistic information when paired with the dominant inflammatory signal of CRP (relative importance 78.65%). Failure to account for thyroid autoimmunity in predictive modeling may obscure early preeclampsia risk in patients presenting with subclinical endocrine vulnerability. Incorporating these subtle endocrine markers into our Naive Bayes model contributed to its high sensitivity (99.9%) and overall accuracy (92.5% ± 7.7%), affirming that joint immune-endocrine profiling outperforms single-biomarker assessments for early risk stratification.

CRP, an inflammatory marker, also emerged as a key predictor of preeclampsia. This finding is consistent with the understanding that preeclampsia involves endothelial disruption and heightened systemic inflammatory activation [14]. The inclusion of CRP in our predictive model underscores the role of inflammation in the pathogenesis of preeclampsia. It suggests that the inflammatory cascade may be a critical component of the disease process, potentially contributing to the hypertension and proteinuria that characterize preeclampsia. This disruption is reflected in altered levels of inflammatory markers such as CRP, an n acute-phase reactant whose levels increase significantly during infection and inflammation [10].

Furthermore, our network analysis revealed a bridging connection between maternal and cord parameters through maternal FT3 and cord TSH. This connection formed the basis of the probabilistic prediction of apparent fetal hypothyroidism through cord blood TSH levels. The accuracy of the NB model for predicting high cord TSH by maternal FT3 was 63.7% ± 5.3% overall and increased to 73.3% ± 1.7% within the preeclampsia group. This suggests that the model's predictive ability is enhanced in preeclampsia, indicating a stronger association between maternal thyroid function and fetal thyroid status.

From a translational perspective, the lightweight, probabilistic architecture of our Naive Bayes model offers potential for practical implementation in resource-constrained and peripheral healthcare settings, such as primary health centers, mobile medical units, and rural clinics in low- and middle-income countries. Unlike computationally intensive deep learning models, Naive Bayes algorithms require minimal processing power. They can be deployed in lightweight mobile applications, offline tablet interfaces, or basic web tools without relying on high-end hardware or continuous internet access. Furthermore, the underlying predictor panel, comprising CRP, TSH, and ATPO, utilizes established, cost-effective biomarkers that can be adapted to point-of-care lateral flow or portable immunoassay testing kits. In peripheral settings where advanced ultrasonography, Doppler imaging, or subspecialty obstetric care are often unavailable, this screening framework serves as a rapid, low-cost risk-triage tool. Frontline healthcare workers can input routine biochemical profiles during antenatal visits to stratify preeclampsia risk early in gestation, facilitating timely risk-informed referrals to tertiary care facilities for specialized maternal-fetal surveillance and proactive management.

This study has several limitations that must be acknowledged when interpreting the findings. The difference in the prevalence of elevated TSH levels between normal and preeclamptic pregnancies was not statistically significant between normal pregnancies and those affected by preeclampsia, potentially attributable to sample size or other confounding variables. The study population may not accurately represent all pregnant women, potentially constraining the external validity of the results. Research with larger, more heterogeneous cohorts is necessary to corroborate our findings and investigate the potential impact of ethnicity and other demographic variables on the model's predictive efficacy. A retrospective audit of data is feasible in a rural region of a low- and middle-income country; however, certain places exhibit data deficiencies [1].

The secondary Naive Bayes model predicting cord blood TSH elevation from maternal FT3 achieved 100% sensitivity but demonstrated a low overall specificity of 27.4%, which improved to 46.7% within the preeclampsia subgroup. In screening paradigms for neonatal endocrine abnormalities, maintaining near-perfect sensitivity is important for minimizing false negatives, as missed cases of fetal/neonatal hypothyroidism carry severe neurodevelopmental risks. However, we acknowledge that a specificity of this magnitude generates a high false-positive rate, rendering the current model unsuitable as a standalone diagnostic tool. Instead, this secondary model serves as an exploratory, low-cost screening framework demonstrating that maternal-fetal thyroid axis coupling appears stronger in preeclamptic pregnancies. Future model iterations must incorporate additional maternal inflammatory indices or Doppler hemodynamic parameters to improve specificity and reduce false positives prior to clinical translation.

## Conclusions

This study used a Naive Bayes network model to predict the risk of preeclampsia and its impact on fetal outcomes. CRP, TSH, and ATPO levels were identified as the most significant indicators of preeclampsia. A strong association was observed between maternal FT3 and cord blood TSH levels. The findings suggest an association between preeclampsia and alterations in fetal thyroid-related parameters. The predictive model demonstrates substantial potential for early risk assessment, which may facilitate timely medical intervention.

Our findings demonstrate that integrating maternal inflammatory and thyroid profiles within a lightweight Naive Bayes architecture achieves high overall performance for preeclampsia risk screening. Systemic inflammation, as reflected by maternal CRP, is the predominant driver of model accuracy, while maternal ATPO and TSH contribute secondary predictive value in the multivariable space. Unsupervised network analysis identified maternal FT3 as a potential cross-compartment link to fetal cord TSH elevation; however, the secondary fetal screening model demonstrated low specificity and high false-positive rates, precluding its use as a standalone diagnostic tool. Future prospective, multicenter studies with larger cohorts are necessary to validate these preliminary findings, refine model specificity, and further evaluate the clinical utility of joint immune-endocrine profiling in gestational hypertensive disorders.
